# Advancing Dental Risk Profiling: A Literature Review of the Cariogram Model

**DOI:** 10.7759/cureus.80069

**Published:** 2025-03-05

**Authors:** Priyanka Paul, Shrivardhan Kalghatgi, Tanushree Dalvi, Sayali Sudhir Magdum

**Affiliations:** 1 Public Health Dentistry, Bharati Vidyapeeth (Deemed to be University) Dental College and Hospital, Sangli, IND

**Keywords:** cariogram, dental caries, microorganism, preventive dentistry, risk assessment tools

## Abstract

The Cariogram is a well-established tool for caries risk assessment, integrating clinical, behavioral, and microbiological factors into an accessible graphical display. This review critically evaluates its performance in preventive dentistry, focusing on reliability, accuracy, and clinical value. It examines the methodological design, explores studies validating its predictive power, and compares its strengths with standard caries risk assessment methods. Additionally, the analysis highlights the tool’s adaptability across diverse populations and settings while acknowledging limitations such as reliance on subjective measures and the demanding nature of data collection. Furthermore, recent studies on integrating digital technologies and artificial intelligence (AI) with the Cariogram are discussed, emphasizing their potential to enhance personalized oral healthcare. Evidence suggests that the Cariogram not only aids clinicians in planning interventions but also educates patients about their caries risk, fostering better adherence to preventive practices. Emerging research on AI-supported applications confirms the Cariogram’s promise in transforming caries risk assessment by making it more practical, scalable, and accessible. This review consolidates current evidence on its role in modern dentistry and explores future directions for optimizing caries risk assessment and prevention.

## Introduction and background

Dental caries is one of the most widespread diseases globally, affecting individuals of all ages, with a higher prevalence among younger populations. Despite the global practice of preventive dentistry, underserved communities continue to experience a significant burden of this condition. To enhance caries prevention beyond conventional management techniques, there is a need for predictive, customized, and evidence-based approaches [1].

In 1996, Bratthall and Petersson introduced the Cariogram model, a caries risk assessment tool designed to predict an individual’s risk and guide personalized preventive strategies [2]. Unlike traditional approaches that rely on isolated risk factors such as bacterial counts or fluoride exposure, the Cariogram integrates multiple determinants of caries development - including dietary habits, oral hygiene practices, bacterial levels, salivary properties, and systemic health conditions - into a single visual output: a pie chart representing the “probability of avoiding caries” [3].

The Cariogram has been widely applied in clinical fields such as geriatrics, orthodontics, and pediatric dentistry. Additionally, epidemiological studies have used it to assess caries risk in specific populations, including diabetic patients and individuals with special medical needs [4]. However, despite its frequent use, certain limitations exist, such as its reliance on laboratory data, salivary flow rate, and bacterial counts and the subjectivity involved in assessing behavioral factors.

Research has demonstrated the Cariogram’s validity and effectiveness in caries risk assessment. Cagetti et al. found a significant correlation between Cariogram outcomes and actual caries experience, confirming its predictive value for adolescents over a two-year period [5]. Similarly, Petersson et al. emphasized its ability to distinguish high-risk from low-risk individuals, allowing for targeted preventive measures [6].

This review aims to examine the Cariogram model’s role in dental practice, exploring its clinical applications, benefits, and limitations.

## Review

Structure of the Cariogram model

Table 1 presents the colored segments of the Cariogram model, each representing different functions based on the patient’s clinical findings.

**Table 1:** Elements of the Cariogram model The Cariogram is displayed as a pie chart divided into five colored sections, each representing a specific aspect of caries risk and preventive capacity [7].

Segment	Function
Green segment	Chance to avoid new caries
Red segment	Actual disease risk
Blue segment	Diet
Dark blue segment	Bacteria
Yellow segment	Susceptibility

This comprehensive tool facilitates caries risk evaluation by visually summarizing key risk factors and offering tailored prevention recommendations. Beyond risk assessment, the Cariogram plays a crucial role in patient education and aids in refining treatment strategies to enhance preventive care [8].

**Figure 1:**
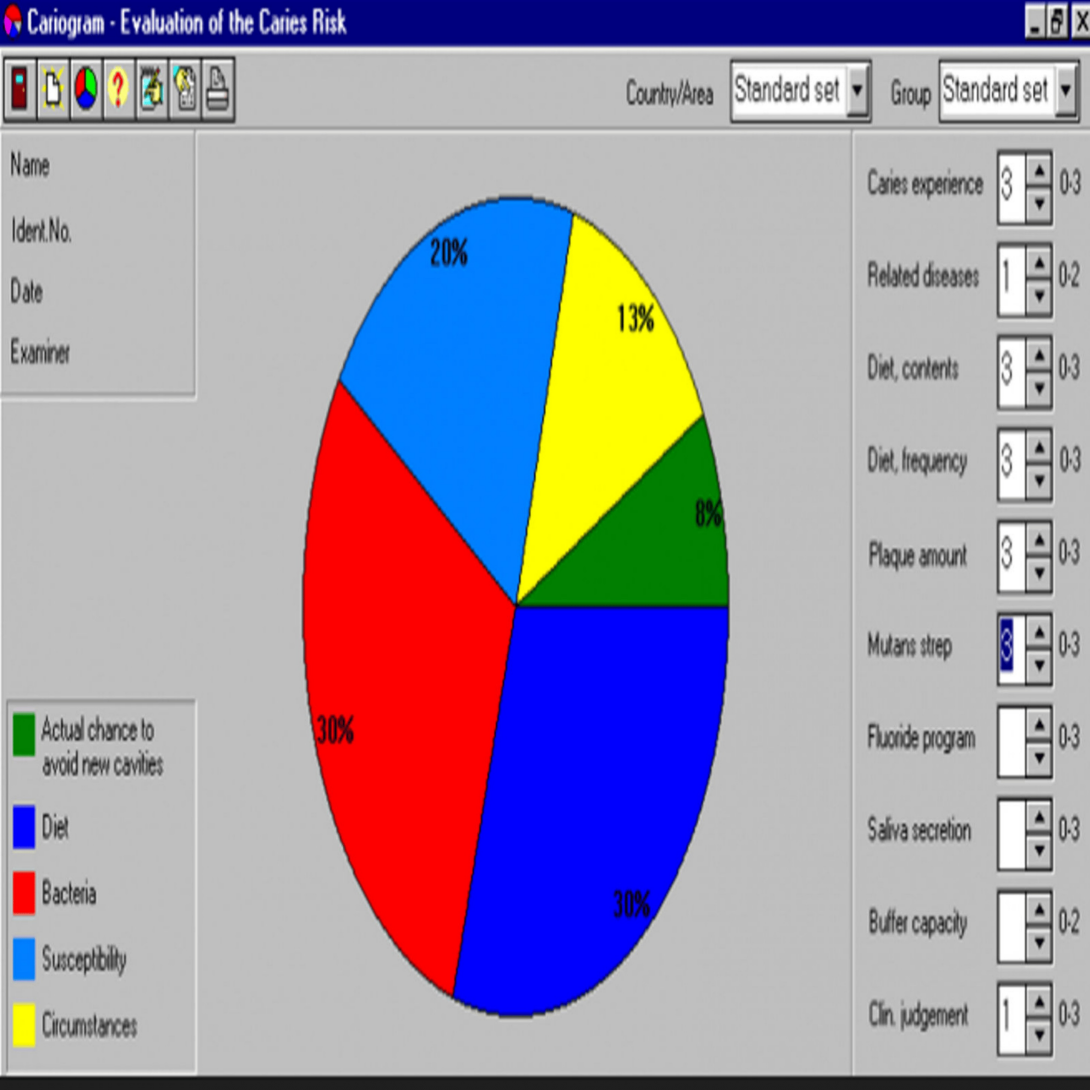
Pie chart representation of the Cariogram model The pie chart provides a graphical representation of the percentage distribution of factors contributing to an individual’s susceptibility to caries, offering a visual overview of risk and preventive capacity.

Figure 1 illustrates the graphical representation of dietary factors, microbial count, chance to avoid caries, susceptibility, and individual circumstances.

Table 2 outlines the factors measured in the Cariogram model that are associated with dental caries. Failure to control these factors increases the risk of caries progression. Uncontrolled sugar intake and poor oral hygiene contribute to higher bacterial production, further elevating caries risk. Early identification of these risk factors can support better oral health outcomes. The Cariogram model provides a simplified and effective preventive approach for maintaining overall oral health. 

**Table 2:** Function of the Cariogram model The Cariogram model includes 10 critical variables that influence caries development, organized into five distinct categories.

Factors	Functions
Dietary factors	Sugar intake frequency: tracks the intake of sugary foods or beverages daily [9]
Bacterial factors	Levels of mutans streptococci and lactobacilli: identified through saliva testing, as these bacteria are primary contributors to enamel demineralization by producing acids [10]
Susceptibility factors	Fluoride exposure: examines fluoride intake from sources like toothpaste, water, or professional treatments
	Salivary flow rate: measured in milliliters per minute; decreased flow increases the risk for caries
	Saliva buffering capacity: refers to the ability of saliva to neutralize acids, with lower buffering capacity associated with increased caries risk
Circumstances	Caries history: a history of caries is a strong predictor of future risk
	Systemic conditions: conditions that increase caries risk by reducing saliva production, such as diabetes

The Cariogram model: visualizing caries risk and prevention potential

The Cariogram generates a pie chart that visually represents a patient’s caries risk and preventive potential. By summarizing complex data in an intuitive format, it enhances understanding for both healthcare professionals and patients [11]. For instance, individuals with good oral hygiene, low sucrose intake, and regular fluoride use will have larger green sections, indicating a lower risk of dental caries and a greater capacity for prevention. Conversely, patients with poor dietary habits, high bacterial levels, and limited fluoride exposure will exhibit more red sections, reflecting a higher caries risk. The Cariogram scores categorize patients into high-, moderate-, or low-risk groups for dental caries [12].

This information helps clinicians tailor preventive strategies based on risk levels: (1) high-risk patients benefit from intensive dietary counseling, antibacterial treatments, and salivary interventions such as salivary stimulants; (2) moderate-risk patients require improved oral hygiene and regular fluoride application; and (3) low-risk patients should maintain existing preventive measures with routine monitoring.

Advantages of the Cariogram model

The Cariogram model offers several advantages in assessing and managing caries risk: (1) Holistic risk assessment: by integrating multiple factors, it provides a comprehensive evaluation, unlike conventional methods that focus on individual risk components. This approach considers the complex interplay between biological, behavioral, and environmental influences [13]. (2) Customized care: the Cariogram supports personalized treatment by identifying key contributors to a patient’s caries risk, allowing for targeted interventions. For instance, a patient with poor dietary habits but adequate saliva flow would benefit more from dietary modifications than from salivary stimulants. (3) Effective patient education: its visual pie chart representation helps patients understand how their lifestyle and habits influence their caries risk, motivating them to adopt healthier oral practices. (4) Tracking and evaluation: the Cariogram enables continuous monitoring of changes in caries risk over time, helping clinicians assess the effectiveness of preventive strategies and adjust treatment plans accordingly [14].

Discussion

Table 3 illustrates the various applications of the Cariogram model in dentistry, a visual and interactive tool used in clinical practice to assess an individual’s caries risk. By comprehensively evaluating factors such as diet, bacterial load, and caries susceptibility, the Cariogram helps in identifying preventive measures to reduce the likelihood of future caries development.

**Table 3:** Applications of the Cariogram model in clinical settings The use of the Cariogram model is a highly efficient approach for assessing caries risk in clinical practice.

Specialty	Application
Pediatric dentistry	The Cariogram is highly effective in assessing caries risk in children, who are more susceptible to dietary and bacterial factors. It helps guide preventive strategies such as fluoride varnish applications and parental education [15].
Orthodontic patients	Orthodontic appliances complicate oral hygiene, increasing the risk of caries. The Cariogram identifies at-risk individuals and facilitates interventions like antibacterial mouthwashes or fluoride applications [16].
Geriatric dentistry	Older adults often experience reduced saliva flow and systemic health challenges, making them more vulnerable to caries. The Cariogram aids in risk assessment and informs interventions such as fluoride treatments or salivary substitutes [17].
Patients with special healthcare needs	Individuals with systemic conditions like diabetes or those undergoing radiation therapy for head and neck cancers benefit from the Cariogram’s ability to evaluate risk factors related to medical conditions and provide individualized preventive recommendations [18,19].

The Cariogram model has been widely utilized in clinical dentistry as an effective method for assessing caries risk. Its primary strength lies in its ability to consolidate multiple risk factors into a single, visual format, making it a valuable tool for both dental professionals and their patients. For instance, a study by Petersson et al. demonstrated that the Cariogram model enhanced dentists’ ability to identify individuals at high risk more effectively than traditional assessment methods [6]. Additionally, presenting risk factors through a visual aid was shown to improve communication with patients, motivating them to adopt preventive measures.

Further validation of the Cariogram model was provided by Utreja et al., who evaluated caries risk among school children [20]. Their findings indicated that the Cariogram accurately predicted caries progression risk, emphasizing the importance of diet and bacterial activity. This study highlighted the model’s potential in guiding preventive interventions, such as dietary modifications and fluoride applications, particularly for high-risk populations. The model has also proven beneficial in pediatric dentistry for evaluating early caries progression through dietary patterns. Several researchers applied it to assess caries risk among Egyptian children, reporting a high degree of accuracy in distinguishing between high-risk and low-risk groups [21]. The study underscored the model’s role in educating parents about the critical importance of maintaining good oral hygiene and a healthy diet for their children.

Beyond clinical applications, the Cariogram has been extensively used in research to assess caries risk across different populations [22]. Vasireddy et al. conducted a longitudinal study examining the relationship between socio-economic factors and caries risk among Hispanics using the Cariogram tool. Their findings revealed a higher caries potential among individuals from low-income backgrounds due to limited access to healthcare and lower fluoride uptake [23]. These results underscore the need to address social determinants of health to enhance caries prevention. Another significant study by Sen et al. evaluated the utility of the Cariogram in assessing caries risk among diabetic patients. The findings confirmed that diabetic individuals exhibited a higher risk of caries due to reduced salivary flow and increased bacterial activity [24-26]. Based on these studies, it was concluded that the Cariogram is a valuable tool for assessing caries risk in medically compromised patients.

Table 4 presents various studies that have incorporated the Cariogram model in dentistry.

**Table 4:** Studies on the utilization of the Cariogram model in dentistry These studies collectively demonstrate the utility and versatility of the Cariogram model in assessing caries risk across diverse age groups, geographic regions, and clinical settings.

Study	Population	Results	Discussion
Cagetti et al. (2022) [5]	School children	The Cariogram identified dietary habits and mutans streptococci levels as significant risk factors.	Supports the model’s applicability in pediatric populations for targeted preventive care
Petsi et al. (2014) [16]	Adolescent orthodontic patients	Cariogram profiles indicated an increased caries risk in patients with orthodontic appliances.	Suggests the need for enhanced preventive measures for orthodontic patients based on Cariogram assessments
Campus et al. (2012) [19]	Italian adolescents	The Cariogram effectively predicted caries increment over a two-year period.	Demonstrates the model’s prognostic value in adolescents for long-term caries management
Giacaman et al. (2013) [25]	Chilean adolescents and adults	The Cariogram effectively assessed caries risk, with higher risk correlating with greater caries experience.	Highlights the model’s utility across different age groups and its potential for guiding preventive strategies
Petersson and Twetman (2015) [27]	Swedish adults	Cariogram assessments correlated with actual caries development over a three-year follow-up.	Validates the model’s predictive capability in adult cohorts
Karamüftüoğlu and Ulusu (2021) [28]	Children	The Cariogram identified high caries risk linked to frequent sugar intake and low fluoride exposure.	Emphasizes the need for dietary counseling and fluoride use in caries prevention strategies
Kavvadia et al. (2012) [29]	Greek preschool children	The Cariogram identified high caries risk associated with low socioeconomic status and poor oral hygiene.	Highlights the need for targeted preventive programs in socioeconomically disadvantaged groups
Su et al. (2021) [30]	Systematic review and meta-analysis	The Cariogram demonstrated moderate predictive performance for caries risk across various studies.	Recommends further validation and potential refinements to improve the model’s accuracy

The impact of the Cariogram extends beyond individual patient care, offering significant public health benefits as well [31-33]. At the individual level, it enables personalized prevention strategies, improving patient outcomes while reducing treatment costs. On a broader scale, it serves as a framework for identifying at-risk populations and developing targeted interventions, such as community-based fluoride programs and oral health education campaigns. One of the Cariogram most significant advantages is its ability to individualize preventive care [32-34].

A study by Zukanovic demonstrated that patients who received their Cariogram scores were more likely to adopt preventive measures, including reducing sugar intake and improving oral hygiene habits [33]. By identifying specific risk factors, clinicians can tailor interventions accordingly. For example, a patient with high bacterial levels but good oral hygiene would benefit most from antibacterial treatments, while one with poor dietary habits would require intensive dietary counseling. The Cariogram has also been shown to enhance patient understanding and compliance by visually representing risk factors, making it a powerful educational tool [34-36].

Moreover, the Cariogram can be adapted for different populations and culturally adjusted, recognizing that caries risk factors vary across regions. Dietary components, in particular, may need to be modified based on regional food habits. Additionally, the model holds potential as an advanced tool for detecting the progression of dental caries in both cavitated and noncavitated lesions, further strengthening its role in caries prevention and management.

Future directions

Future developments of the Cariogram model could focus on enhancing its accessibility, applicability, and comprehensiveness. Simplifying the model for low-resource settings by integrating basic clinical assessments and self-reported data could provide a practical approach to risk evaluation, enabling community-based interventions and preventive strategies. Additionally, incorporating digital tools and developing a version that does not rely on laboratory testing could improve its usability in resource-limited environments. Proxy measures, such as self-reported salivary flow and bacterial levels, could serve as alternatives to laboratory-based data, making the tool more accessible. Furthermore, expanding the Cariogram to assess other oral health conditions, such as periodontal disease or oral cancer, could offer a more comprehensive evaluation of overall oral health, increasing its clinical utility [37-39].

## Conclusions

The Cariogram model is a well-established risk assessment tool that can be effectively utilized in low-resource settings to evaluate the burden of dental caries in populations. Its integration into clinical practice and dental health education systems is essential for providing valuable insights into caries prevention, enabling more targeted and effective preventive strategies.
